# Management of psoriasis-like rash associated with idelalisib monotherapy in a patient with refractory follicular lymphoma: a case report

**DOI:** 10.1186/s13256-020-2344-9

**Published:** 2020-02-24

**Authors:** Salma Machan, Carlos Plaza, Yosmar Pérez-González, Maria Rodriguez-Pinilla, Luis Requena, Raul Cordoba

**Affiliations:** 1 Department of Dermatology, Fundación Jiménez Díaz University Hospital, Health Research Institute IIS-FJD, Madrid, Spain; 2 Lymphoma Unit, Department of Hematology, Oncohealth Institute, Fundación Jimenez Diaz University Hospital, Health Research Institute IIS-FJS, Autonomous University of Madrid, Madrid, Spain; 3 Department of Pathology, Fundación Jiménez Díaz University Hospital, Health Research Institute IIS-FJD, Madrid, Spain

**Keywords:** Adverse drug event, Cutaneous drug reaction, Case report, Follicular lymphoma, Idelalisib, Rash, Psoriasis-like, Psoriasiform

## Abstract

**Background:**

Follicular lymphoma is an indolent non-Hodgkin lymphoma that is most commonly diagnosed in elderly individuals. The majority of patients with follicular lymphoma present with advanced disease. Despite the recent advances in treatment, there remains a substantial unmet need for effective treatments for patients with relapsed/refractory follicular lymphoma. The PI3Kδ inhibitor idelalisib was approved by the European Medicines Agency in 2014 as a monotherapy for the treatment of adult patients with follicular lymphoma that is refractory to two prior lines of treatment. Real-world evidence from patients with follicular lymphoma treated with idelalisib indicates its utility in these patients.

**Case presentation:**

This case report describes an 82-year-old, retired, white, female patient with refractory follicular lymphoma who achieved a partial response with idelalisib treatment. Despite experiencing two incidences of a psoriasis-like rash during idelalisib treatment that required effective management with topical steroids, the patient was able to restart treatment successfully and maintain a continued partial response.

**Conclusions:**

The clinical relevance of the effective management of adverse events in this case demonstrates the opportunity to enable patients to remain on therapy, thereby maintaining long-term response and improving overall outcomes.

## Background

Follicular lymphoma (FL) is an indolent non-Hodgkin lymphoma that is most often diagnosed in elderly individuals, and most patients present with advanced disease [1]. Despite recent treatment advances, such as anti-CD20 monoclonal antibodies, alkylating agents, and radioimmunotherapy, there remains a substantial unmet need for effective treatments for patients with relapsed/refractory FL [1]. As a result, idelalisib received accelerated Food and Drug Administration approval and European Medicines Agency approval in 2014, based on the phase 2, single-arm, multicenter trial (*n* = 72) that evaluated the safety and efficacy of idelalisib monotherapy in patients with FL who had relapsed within 6 months following treatment with rituximab and an alkylating agent and ≥2 prior treatments [2, 3]. Idelalisib is indicated in Europe as a monotherapy for the treatment of adult patients with FL that is refractory to two prior lines of treatment [2]. In the United States, idelalisib is indicated for the treatment of patients with FL who have received at least two prior systemic therapies [4]. According to treatment guidelines and expert opinion, idelalisib has demonstrated significant efficacy and an acceptable safety profile in clinical trials for patients with double-refractory FL [5, 6]. Clinical trials have reported that ≤ 18–21% of patients receiving idelalisib for the treatment of indolent non-Hodgkin lymphoma and chronic lymphocytic leukemia developed all-grade rash, and 2–3% of patients experienced grade ≥ 3 rash [7]. However, real-world experience with idelalisib in patients with FL is highly valuable in addition to evidence from clinical trials in order to optimize patient management in day-to-day clinical practice.

In this case report, we describe a case of an elderly patient with refractory FL who was initiated on idelalisib and achieved a partial response, experienced two incidences of a psoriasis-like rash during idelalisib treatment that required effective management, and was able to restart treatment successfully.

## Case presentation

### Presenting concerns

**Fig. 1:**
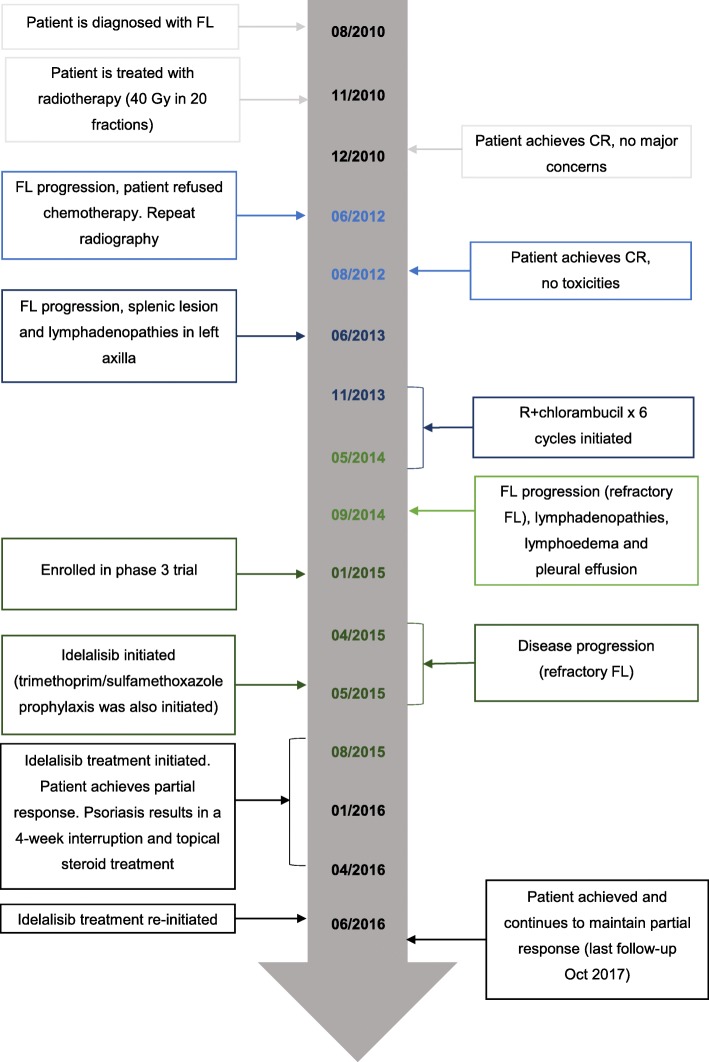
Timeline of patient history of treatments. *CR* complete response, *FL* follicular lymphoma

The current case report describes a retired, white, female patient who was 82 years old and from Spain. She was diagnosed with FL in August 2010 and presented with lymphadenopathy in the right femoral region with FL, grade 3A, a Follicular Lymphoma International Prognostic Index risk score of 1, low tumor burden, and no bone marrow involvement. A timeline of the patient history, interventions, and clinical findings is shown in Fig. 1. The patient had no relevant prior medical history. She was treated with radiotherapy (40 Gy in 20 fractions) for stage I localized disease and achieved a complete response (CR) with no major concerns.

In June 2012, approximately 18 months after CR was achieved, the patient experienced FL progression, presenting with a submandibular mass. A biopsy revealed grade 3a, stage Ia FL. The patient refused chemotherapy at that time, so she was treated with repeat radiotherapy (40 Gy in 20 fractions) and achieved CR with no toxicities. However, 9 months following the second CR, the patient experienced FL progression, presenting with lymphadenopathies in the left axillar region and splenic lesions, and biopsy revealed grade 1, stage IIIa FL. She was treated with six cycles of rituximab-chlorambucil in lieu of more toxic treatment options that the patient had refused. She achieved a partial response and refused further treatment at that time. Four months following the last treatment, the patient experienced FL progression (refractory FL) and presented with lymphadenopathies in the left axillar region and grade 3/4 lymphedema in the left arm. Biopsy revealed grade 1 FL. The patient also showed left pleural effusion (not investigated further in this case study). She was enrolled in a randomized, double-blind, phase 3 study evaluating rituximab in combination with an investigational therapy versus rituximab and placebo. The patient progressed after 4 months.

Based on the refractory nature of the disease following two lines of chemoimmunotherapy (including an immunomodulatory drug treatment), a fourth relapse, and disease that was refractory to both rituximab and chlorambucil, the decision was made to initiate the patient on idelalisib monotherapy (150 mg orally twice daily). Trimethoprim/sulfamethoxazole prophylaxis was also initiated. Palliation was the alternative treatment option that was considered at this stage. The goal of treatment was resolution of lymphedema and dyspnea.

### Follow-up and outcomes

**Fig. 2:**
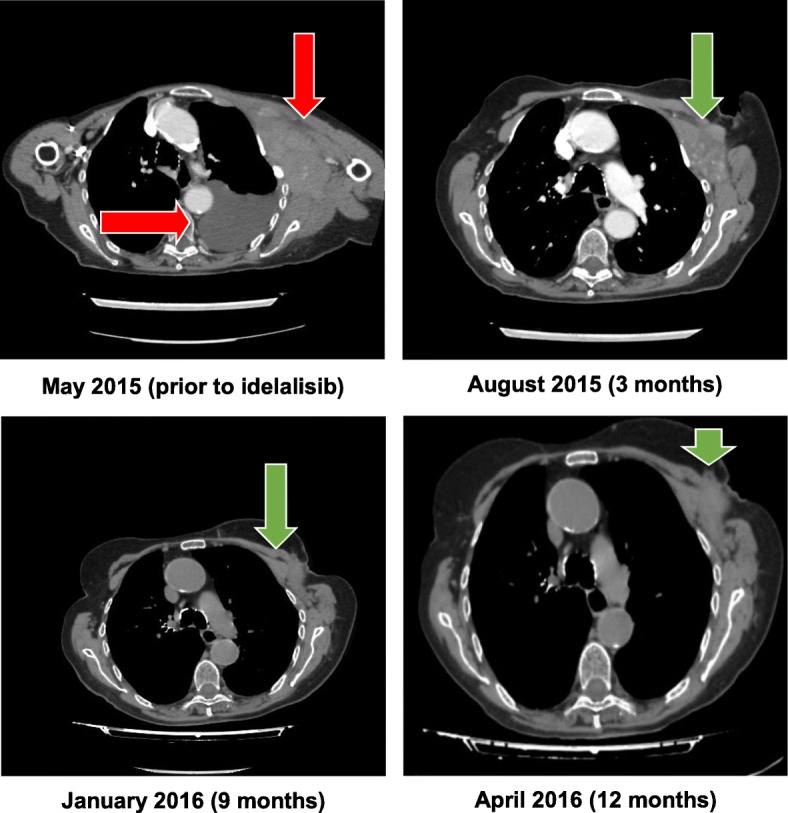
Computed tomography response to idelalisib treatment. Arrows indicate presence of lymphoedema and/or pleural effusion

**Fig. 3:**
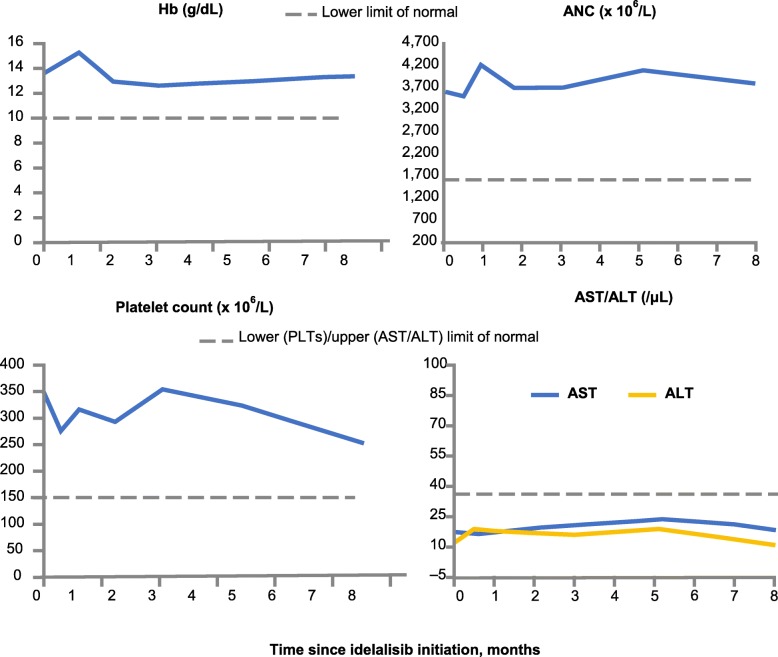
Hematological and liver measures during treatment with idelalisib. *ANC* absolute neutrophil count, *ALT* alanine aminotransferase, *AST* aspartate aminotransferase, *Hb* hemoglobin, *PLT* platelet

Response to idelalisib treatment (started on 19 May 2015) was observed at the 3-month follow-up in this patient, as indicated by the computed tomographic scans shown in Fig. 2. After 3 months of treatment, there was a significant reduction in lymphedema in the left arm, a partial response of the lymph nodes according to Lugano criteria [8], and clearance of pleural effusion. At 6 months, the remaining lymphedema in the left arm was almost entirely resolved, and she remained in partial response (almost reaching CR) at the 9-month follow-up, with no evidence of pleural effusion. At 12 months, following initiation of treatment with idelalisib, the patient demonstrated a sustained partial response (almost CR), continued to have no pleural effusion, and lymphedema was resolved. Overall, the patient tolerated idelalisib well and reported good adherence to treatment. There were no hematological concerns or liver toxicity observed following the initiation of idelalisib. Hemoglobin, absolute neutrophil counts, platelet counts, and aspartate aminotransferase/alanine aminotransferase levels all remained within normal limits throughout treatment (Fig. 3).

**Fig. 4:**
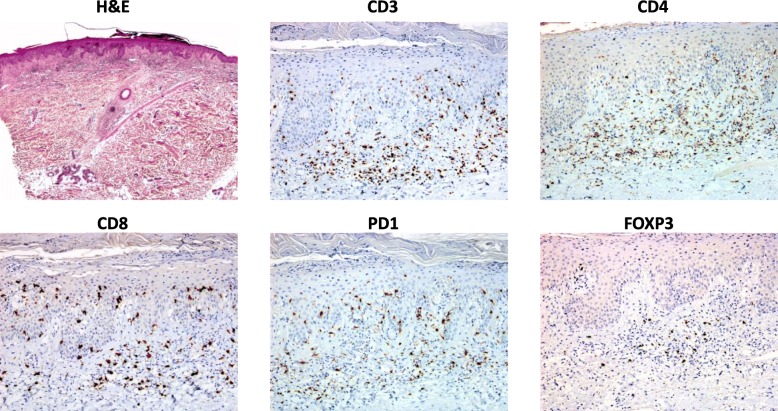
Histopathology of skin biopsy of Grade 2 psoriasis-like rash occurring 11 months after idelalisib initiation. Haemotoxylin and eosin (H&E) stain showed psoriasiform epidermal hyperplasia with subcorneal pustule and mild perivascular superficial lymphocytic infiltrate, which expressed CD3, CD4, CD8 and PD1, with few cells expressing FOXP3 immunoreactivity

**Fig. 5:**
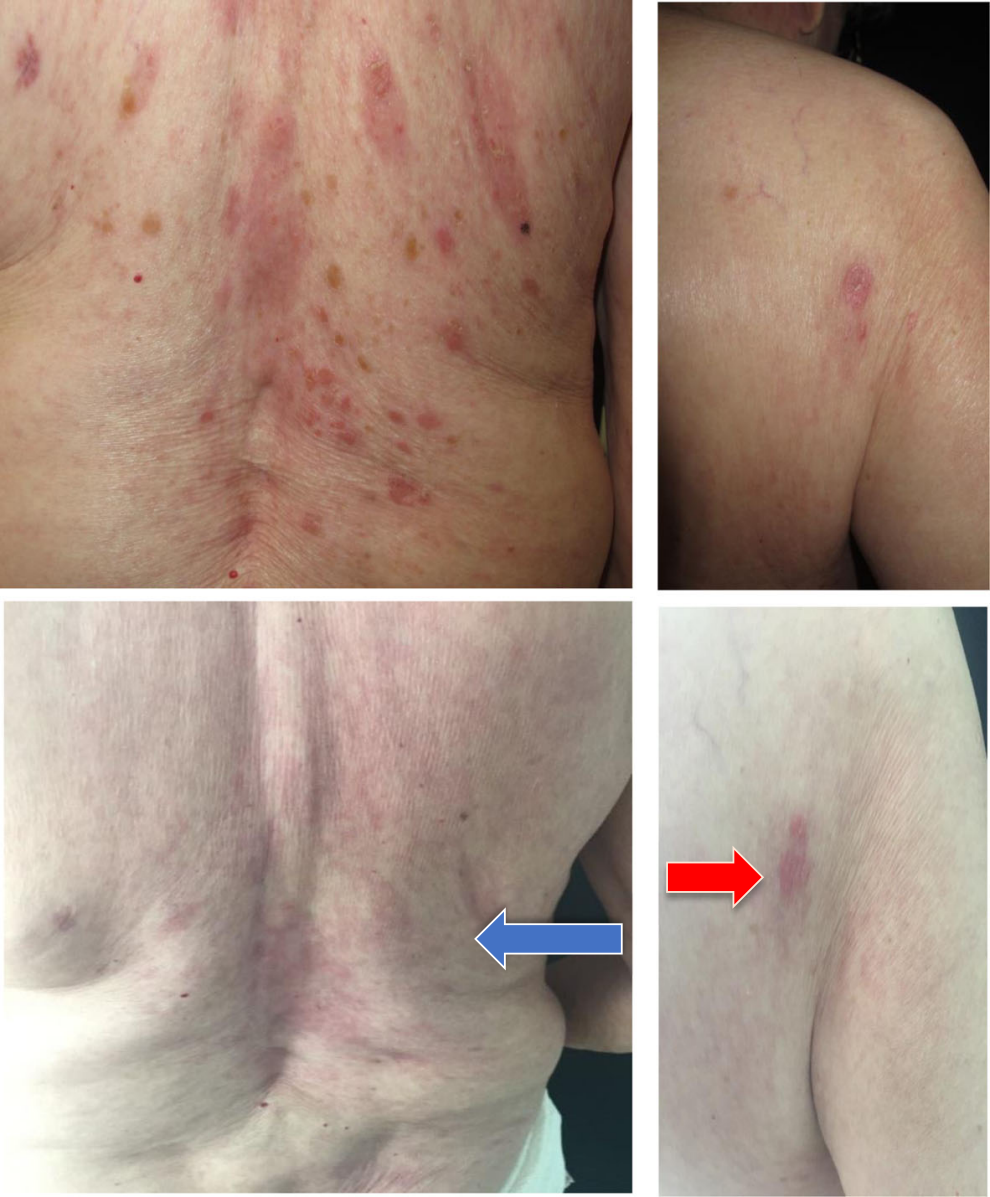
Grade 2 psoriasis-like rash consisting of erythematosquamous papules and plaques, occurring 11 months after idelalisib initiation. (**a**, **c**) Treatment with topical steroids and 4-week interruption of idelalisib dosing. (**b**, **d**) Improvement without resolution after topical steroids and 4-week interruption of idelalisib dosing. *Blue arrow* indicates psoriasis-like rash that has been partially resolved. *Red arrow* indicates persistent erythematosquamous plaque

After 11 months of treatment with idelalisib, the patient developed erythematosquamous papules and plaques, with some pustules at the periphery limited to the scalp, left forehead, back, buttocks, and over some scars on the abdomen and the right side; without accompanying symptoms. This was designated a grade 2 rash that was described as psoriasis-like, with T-cell infiltration based on skin histopathology (Fig. 4). In line with suggested guidance, idelalisib dosing was interrupted for 4 weeks [2], and the patient was treated with topical steroids. She experienced improvement to grade 1 but without complete resolution (Fig. 5). Idelalisib was reinitiated at a lower dose of 100 mg twice daily. A physical examination revealed that the patient did not experience any worsening of any skin lesions, lymphoedema, and no palpable lymphadenopathy in the left axillar region after 1 month from re-initiating treatment. However, the patient then experienced a second recurrence of the psoriasis-like rash, which led to a second interruption of treatment for 5 weeks. These symptoms were well managed with topical steroids, and the patient then received a reduced dose of idelalisib 100 mg twice daily without a further recurrence.

The patient continued on idelalisib treatment and maintained a partial response up to the most recent visit (in October 2017), thereby demonstrating a maintained partial response over 30 months. No lymphoma progression was observed during the interruptions of treatment.

## Discussion and conclusions

This case report demonstrates that treatment with idelalisib led to a rapid and durable response in an elderly patient with double-refractory FL, despite disease progression with prior treatment, including radiotherapy, immunomodulatory therapies, and alkylating agents. The patient experienced effective control of symptoms such that lymphedema was almost entirely resolved and pleural effusion was completely resolved. Treatment with idelalisib over the first 10 months was well tolerated with no reported toxicities. The patient presented with a psoriasis-like rash after 11 months of idelalisib treatment, but with interruption of idelalisib and topical steroids, the rash was well managed so that the patient was able to resume idelalisib therapy and maintain the partial response. Occurrence of, and successful management of, psoriasis-like rash has not been reported previously in association with idelalisib use in patients with FL.

Severe or life-threatening cutaneous reactions (grade ≥ 3) have been reported previously with idelalisib, including exfoliative dermatitis, rash, erythematous rash, generalized rash, macular rash, maculopapular rash, papular rash, pruritic rash, exfoliative rash, and skin disorder [2, 7]. Rash associated with idelalisib usually presents as a flat red area of the skin (on the trunk and extremities), and severity is generally mild-to-moderate and rarely results in discontinuation of treatment [2]. Most reported cases in clinical trials were successfully treated with antihistamines or topical/oral steroids [7]. A phase 2, multicenter, interventional, single-arm study in patients with indolent non-Hodgkin lymphoma (*n* = 125) treated with idelalisib monotherapy reported that up to 13% of patients developed all-grade rash. Up to 2% of these reported rashes were grade ≥ 3, none of which led to discontinuation of study drug [3]. Of the 72 patients with FL included in this trial, two experienced grade ≥ 3 rash, both of whom had dose interruptions, and one discontinued treatment [9]. Similarly, an integrated analysis of eight idelalisib clinical trials reported all-grade rash in 17% of patients, 2% of which were grade ≥ 3. This analysis also revealed that dose interruption in response to adverse events resulted in successful re-challenge in most patients [6].

The patient in this case report developed a grade 2 psoriasis-like rash 11 months after initiation of idelalisib. The suggested management strategy for the development of grade 3–4 rash includes interruption of dosing. When the rash has returned to grade ≤ 1, it is suggested that the patient can continue idelalisib dosing at the reduced dosage of 100 mg twice daily. If rash does not recur, the patient can then resume the standard 150-mg twice-daily dosage [2]. In the case of this patient, interruption of idelalisib dosing combined with topical steroid treatment effectively improved and managed the symptoms of psoriasis-like rash such that idelalisib treatment could be reinitiated. Conversely, in other case descriptions, idelalisib has not been reinitiated after resolution of rash [10, 11]. Here, continued treatment resulted in a maintained partial response, with no evidence of lymphoma progression during the period of dose interruption.

The clinical importance of effective management of adverse events is apparent in the opportunity to enable patients to remain on therapy. By providing this opportunity, patients responding to treatment can maintain this response in the long term, improving overall outcomes.

## Data Availability

The datasets used and/or analyzed during the current study are available from the corresponding author on reasonable request.

## Abbreviations

ANC: Absolute neutrophil count
ALT: Alanine aminotransferase
AST: Aspartate aminotransferase
CR: Complete response
FL: Follicular lymphoma
Hb: Hemoglobin
PLT: Platelets
